# A comparative morphology of the male genitalia of Aphididae (Insecta, Hemiptera): part 2

**DOI:** 10.1007/s00435-012-0163-2

**Published:** 2012-06-26

**Authors:** Karina Wieczorek, Bartosz J. Płachno, Piotr Świątek

**Affiliations:** 1 Department of Zoology, University of Silesia, Bankowa 9, 40-007 Katowice, Poland; 2 Department of Plant Cytology and Embryology, Jagiellonian University, Grodzka 52, 31-044 Cracow, Poland; 3 Department of Animal Histology and Embryology, University of Silesia, Bankowa 9, 40-007 Katowice, Poland

**Keywords:** External reproductive system, Parameres, Phallus, Insects, SEM

## Abstract

The present study provides new data related to the morphology of the male genitalia of Aphididae. The structure of the male genitalia of 39 species from 23 genera of Aphididae was studied using light and scanning electron microscopy. In the species studied, the genitalia of males consist of a phallus composed of the sclerotized basal part with its articulation and a membranous apical part—an aedeagus as well as parameres. This state probably represents the hypothetical plesiomorphic condition of the external male genitalia of aphids. According to the results of the present study, the male genitalia vary among subfamilies (the most varied in Lachninae). Both the phallus and parameres show great variability in their form and the number of setae and may provide characters of taxonomic and diagnostic importance. The shape, size, and modification of parameres are considered in conjunction with the phylogenetic relationships among the studied taxa. Compared with Lachninae, Greenideinae, Aiceoninae, the external genitalia of Aphidinae are less specialized, having many features in common with those of drepanosiphine aphids and differing little from the hypothetical condition. In dwarfish males of Anoeciinae, Thelaxinae, Hormaphidinae, and Eriosomatinae, the miniaturization of the body size affects on the modification of genitalia, mostly parameres. However, the homology of non-modified and modified structures of parameres is not clear.

## Introduction

The male genitalia have proven to be of considerable taxonomic value for various groups of insects. This paper continues our studies into comparative morphology and ultrastructure of the internal and external male genitalia of Aphididae (Insecta, Hemiptera) (Wieczorek 2006, 2008; Wieczorek and Świątek 2008, 2009; Wieczorek et al. 2011). In comparison with other hemipterans, the external male genitalia of aphid species are characterized by simplicity; however, they are well developed and typically consist of the phallus composed of the sclerotized basal part with its articulation and a membranous apical part—the aedeagus. Laterally and above the phallus, there is a pair of setose parameres. In the first part of our studies devoted to external male genitalia (Wieczorek et al. 2011), the general morphologies of these structures were described and compared in those representatives of the Aphididae in which the genitalia are not modified—Drepanosiphinae, Chaitophorinae, Calaphidinae, Phyllaphidinae, Saltusaphidinae, Lizeriinae, Spicaphidinae, Tamalinae, Parachaitophorinae, Phloeomyzinae, and Aphidinae.

In the present study, strongly modified structures of the male genitalia in dwarfish males of the Mindarinae, Hormaphidinae, Anoeciinae, Thelaxinae, and Eriosomatinae as well as in normal-sized males of the Greenideinae, Aiceoninae, and Lachninae are described. To date, the external male genitalia of species belonging to these groups have only been studied in a limited number of species [e.g., *Eriosoma lanigerum* (Hausmann, 1802) (Baker 1915; de Fluiter 1931); *Thelaxes dryophila* (Schrank, 1801) (Polaszek 1987a); *Greenidea* (*Trichosiphum*) *anonae* (Pergande, 1906) (Chaudhuri 1956); *Aiceona japonica* Takahashi, 1960 (Takahashi 1960); *Stomaphis* species (Sorin 1965); *Cinara* species (Eastop 1972); *Eutrichosiphum* *assamense* A. K. Ghosh, R. C Basu & D. N. Raychaudhuri, 1969 (Chakrabarti and Maity 1980)]. They have usually been poorly described or simply illustrated. Only Polaszek (1987b in lit.) provided a comprehensive study of the male genitalia of the entire family Aphididae, using numerous examples from most of these subfamilies.

In this study, we present the general morphologies of the modified male genitalia of selected species of Aphididae, mainly the variability of parameres in their form and location, and consider these structures in conjunction with the phylogenetic relationships among the entire family.

## Materials and methods

### Taxon sampling

The structure of the male genitalia of 39 species belonging to the Mindarinae, Hormaphidinae, Anoeciinae, Thelaxinae, Eriosomatinae, Greenideinae, Aiceoninae, and Lachninae was studied using light (LM) and scanning electron microscopy (SEM). All of the species studied were borrowed from the Muséum national d’Histoire naturelle, Paris, France, with the exception of *Pachypappa tremulae* (Linne, 1761), *Prociphilus fraxini* (Fabricius, 1777), *Anomalosiphum mendeli* Quednau & Martin 2006, and *Schoutedenia ralumensis* Rübsaamen, 1905, which were borrowed from the Natural History Museum, London, United Kingdom. The adult males of *Glyphina betulae* (Linne, 1758), *Lachnus roboris* (Linne, 1758) and nymphs of *Stomaphis quercus* (Linne, 1758) were collected in Poland in the years 2008–2011. Because of the rarity of males, sample sizes consisted of 1–10 individuals. The genitalia of 39 species were studied from aphid material preserved in alcohol and slide-mounted specimens (LM); 13 species were also observed using SEM techniques. The collection data and the microscopic techniques used are summarized in Table 1.

**Table 1:** Collection data for species included in the study

Subfamily/tribe/species	Collection locality	Host plants	Number of studied specimens	LM	SEM
Mindarinae					
*Mindarus abietinus* Koch, 1857	Brouvelieures, France	*Abies pectinata*	10	+	+
*M. japonicus* Takahashi, 1931	Osaka, Japan	*Cephalotaxus drupacea*	2	+	
Hormaphidinae: Nipponaphidini					
*Neothoracaphis yanonis* (Matsumura, 1917)	Matsuzaka, Japan	*Distylium* sp.	3	+	
*Nipponaphis distyliicola* Monzen, 1934	Ise, Japan	*Distylium racemosum*	1	+	
*Monzenia globuli* (Monzen, 1934)	Osaka, Japan	*Distylium* sp.	4	+	
Anoeciinae					
*Anoecia vagans* (Koch, 1856)	Ailli, France	*Cornus sanguinea*	1	+	+
*A. corni* (Fabricius, 1775)	Le Fayet, France	*Cornus* sp.	10	+	+
Thelaxinae					
*Glyphina betulae* (Linne, 1758)	Katowice, Poland	*Betula* sp.	10	+	+
*G. jacutensis* Mordvilko, 1931	St. Etienne-de-Tinee, France	*Alnus incana*	1	+	
Eriosomatinae: Eriosomatini					
*Tetraneura (Tetraneurella)*	Pont de l’Isere	*Ulmus* sp.	2	+	+
*nigriabdominalis* (Sasaki, 1899)	Drome, France				
Eriosomatinae: Pemphigini					
*Pemphigus versicarius* Passerini, 1861	Orange, France	*Colutea arborescens*	4	+	+
*Pachypappa tremulae* (Linne, 1761)	Aspley Heath, UK	*Picea abies*	2	+	
*Prociphilus fraxini* (Fabricius, 1777)	Istanbul, Turkey	*Fraxinus* sp.	9	+	
*P. ligustrifoliae* (Tseng&Tao, 1938)	Hakusan, Japan	*Ligustrum lucidum*	4	+	
Greenideinae: Greenideini					
*Greenidea (Trichosiphum) carpini* Takahashi, 1963	Tokyo, Japan	*Carpinus* sp.	3	+	
*G. (T.) okajimai* Suenaga, 1943	Osaka, Japan	*Shiia* sp.	2	+	
*Eutrichosiphum sinense* D. N. Raychaudhuri, 1956	Kobe, Osaka, Japan	*Shiia* sp.	6	+	
*Mollitrochosiphum (Metatrichosiphon) yamabiwae* Suenaga, 1943	Ise, Japan	*Meliosma rigida*	1	+	
Greenideinae: Cervaphidini					
*Anomalosiphum mendeli*	Sabah, Borneo, Malaysia	–	1	+	
Quednau&Martin 2006					
Greenideinae: Schoutedeniini					
*Schoutedenia ralumensis* Rübsaamen, 1905	Mosman, Australia	*Breynia oblongifolia*	1	+	
Aiceoninae					
*Aiceona japonica* Takahashi, 1960	Kasugayama, Japan	*Cinnamomum camphora*	1	+	
Lachninae: Lachnini					
*Lachnus pallipes* (Hartig, 1841)	Bouvieres, France	*Quercus* sp.	10	+	+
*L. roboris* (Linne, 1758)	Katowice, Poland	*Quercus* sp.	10	+	+
*Pterochloroides persicae* (Cholodkovsky, 1899)	Esfahan, Iran	*Prunus* sp.	1	+	
*Stomaphis aceris* Takahashi, 1960	Shikoku, Japan	*Acer* sp.	4	+	
*S. graffi* Cholodkovsky, 1894	France	*Quercus* sp.	2	+	
*S. pini* Takahashi, 1920	Mt. Shimaji, Japan	*Pinus densiflora*	2	+	
*S. quercus* (Linne, 1758)	La Guyarde, France	*Juglans regia*	10	+	+
	Katowice, Poland	*Quercus* sp.			
*S. yanonis* Takahashi, 1918	Osaka, Japan	*Celtis sinensis*	1	+	
*Maculolachnus submacula* (Walker, 1848)	La Varenne, France	*Rosa* sp.	8	+	+
*Schizolachnus piniradiatae* (Davidson, 1909)	Orono, USA	*Pinus* sp.	5	+	
Lachninae: Eulachnini					
*Eulachnus agilis* (Kaltenbach, 1843)	Haute Alpes, France	*Pinus uncinata*	10	+	+
*E. rileyi* (Williams, 1911)	Haute Alpes, France	*Pinus nigra*	10	+	+
*Cinara cedri* Mimeur, 1936	La Varenne, France	*Cedrus atlantica*	6	+	+
*C. laricis* (Hartig, 1839)	Haute Alpes, France	*Larix europaea*	2	+	
*C. maculipes* Hille Ris Lambers 1966	Mouree, Pakistan	*Pinus excelsa*	7	+	
*C. piceicola* (Cholodkovsky, 1896)	Haute Savoie, France	*Picea excelsa*	7	+	
*C. todocola* (Inouye, 1936)	Tokyo, Japan	*Pinus* sp.	3	+	
*C. (Cedrobium) laportei* (Remaudiere, 1954)	Ifrane, Marocco	*Cedrus atlantica*	4	+	

### Light and electron microscopy

Alcohol-preserved specimens and slides were examined and photographed using Nikon Eclipse 600 and Leica DMR light microscopes. Drawings were made with a camera lucida. A magnified view is provided for each of the photographs and drawings.

The procedure for preparing samples for SEM was as described earlier (Płachno and Świątek 2009, 2010). Briefly, whole specimens were fixed with 2.5 % glutaraldehyde in a 0.1 M phosphate buffer (pH 7.4) for several days or fixed in 70 % ethanol. Later, the samples, which had been dehydrated in ethanol as well as an acetone series, were critical-point dried in liquid CO_2_ and coated with gold using a JEOL-JFC 1100E sputter coater. The specimens were viewed using a HITACHI S-4700 microscope (Scanning Microscopy Laboratory of Biological and Geological Sciences, Jagiellonian University) at 20 kV.

## Results

### Morphology

The external male genitalia of Aphididae consist of the phallus, which is composed of the partially sclerotized basal part with its articulation (sclerotized arms) and a membranous apical part, the aedeagus, as well as parameres. These structures are defined in the following way.

The phallus is the median intromittent organ as a whole.

The basal part of the phallus is a pair of partially sclerotized, various-shaped lobes which enfold the aedeagus and take part in everting and maintaining it in position during copulation. The sclerotized arms of the basal part of the phallus are a pair of sclerotized projections divided into distal and proximal part, giving support for the basal part of the phallus.

The aedeagus is a membranous, copulatory part of the phallus, which is withdrawn within the body and everted during copulation.

The parameres is usually a pair of various-shaped, setose ventral processes positioned at the anterior end of the genital area. In some species of Aphididae, the parameres are strongly modified: completely fused into a single, sharply pointed structure (Anoeciinae, Thelaxinae), reduced to elongated projections supported by additional sclerotization (Eriosomatinae), divided into lobate parts which arise into various-shaped projections toward base of the phallus (Greenideinae, Aiceoninae and partially Lachninae).

The general morphologies of these structures of selected species of Aphididae were described and individual species were then studied systematically (the classification of Aphidoidea is after Nieto Nafria et al. 1998)

#### Mindarinae

**Fig. 1:**
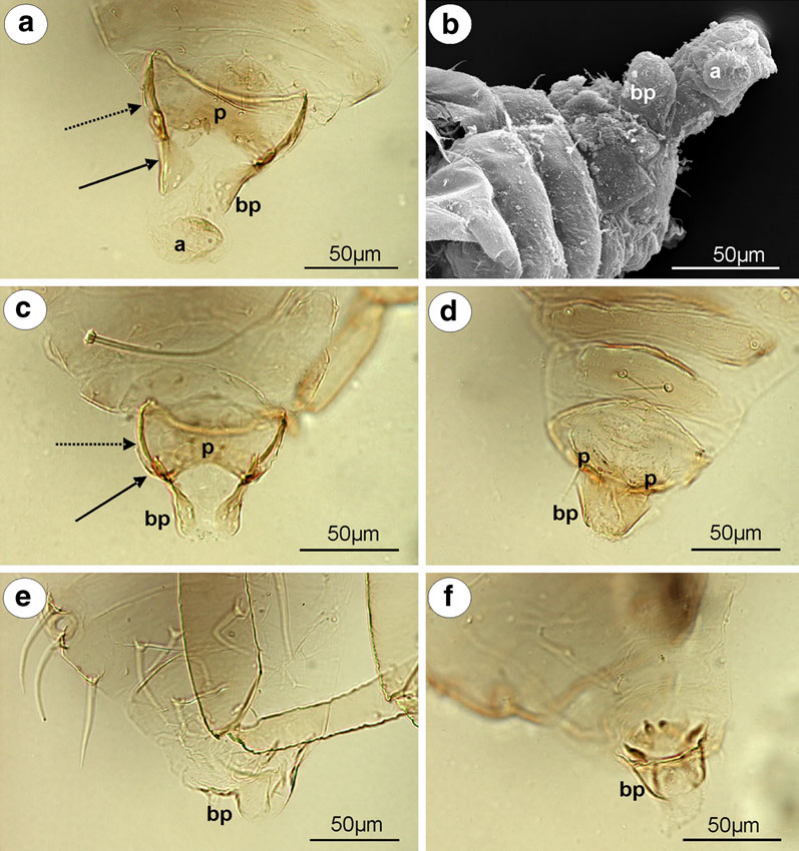
External genitalia of males. **a, b** *Mindarus abietinus*. **a** ventral view (light microscopy), **b** lateral view (scanning electron microscopy). **c** *M*. *japonicus* ventral view (light microscopy). **d** *Neothoracaphis yanonis* ventral view (light microscopy). **e** *Nipponaphis distyliicola* lateral view (light microscopy). **f** *Monzenia globuli* dorsal view (light microscopy). *a* aedeagus, *bp* basal part of phallus with sclerotized arms consists of short proximal (*solid arrow*) and long distal (*dotted arrow*) part, *p* parameres

*Mindarus abietinus* Koch, 1857 (male wingless, dwarfish)—parameres are present, located above the basal part of the phallus, pale, small, basally fused, elongated, with a few setae on the outer margin (Fig. 1a). The basal part of the phallus in slide-mounted specimens (Fig. 1a) and scanning electron microscope (Fig. 1b) is clearly visible, pale, triangular. Sclerotized arms are dark pigmented, with a long distal part and a very short proximal part. The aedeagus is rather short and oval in shape (Fig. 1b).

*M. japonicus* Takahashi, 1931 (male wingless, dwarfish)—external genitalia are very similar to those of *M*. *abietinus* with a club-shaped the basal part of phallus (Fig. 1c).

#### Hormaphidinae

*Neothoracaphis yanonis* (Matsumura, 1917) (male wingless, dwarfish)—parameres are located above the basal part of the phallus, very small and hardly visible in slide-mounted specimens, with a few, short setae on the outer margin. The basal part of the phallus is clearly visible, pale, and forceps-like, without setae. The sclerotized arms are rather short, of a similar length to the proximal and distal parts. They are very thin and pale (Fig. 1d). The aedeagus is not visible.

*Nipponaphis distyliicola* Monzen, 1934 (male wingless, dwarfish)—parameres are hardly visible. The basal part of phallus is clearly visible, pale and lobe-shaped, without setae (Fig. 1e). The sclerotized arms and the aedeagus are not visible.

*Monzenia globuli* (Monzen, 1934) (male wingless, dwarfish)—parameres are hardly visible. The basal part of the phallus is clearly visible, pale and claw-like, without setae (Fig. 1f). The sclerotized arms and the aedeagus are not visible.

#### Anoeciinae

**Fig. 2:**
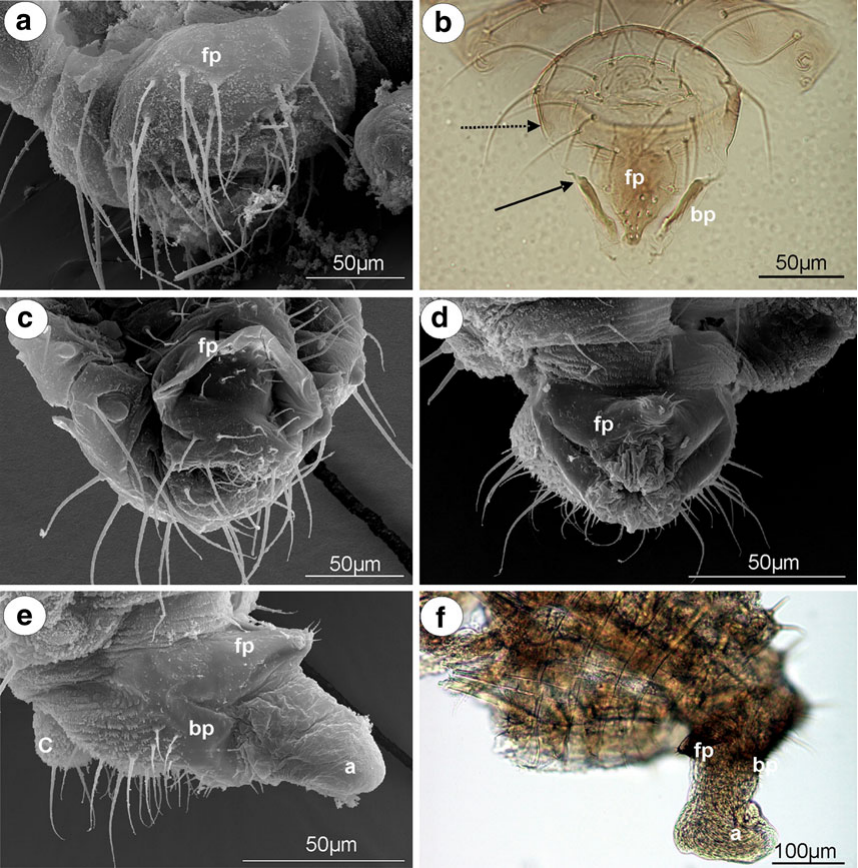
External genitalia of males. **a, b** *Anoecia vagans*. **a** ventral view (scanning electron microscopy), **b** dorsal view (light microscopy). **c** *Anoecia corni* ventral view (scanning electron microscopy). **d**, **e**, **f** *Glyphina betulae*. **d** ventral view (scanning electron microscopy), **e** lateral view (scanning electron microscopy), **f** lateral view (light microscopy). *a* aedeagus, *bp* basal part of phallus with sclerotized arms consists of short proximal (*solid arrow*) and long distal (*dotted arrow*) part, *c* cauda, *fp* fused parameres

*Anoecia vagans* (Koch, 1856) (male wingless, dwarfish)—parameres are present, located above the basal part of the phallus, strongly modified, completely fused into a single triangular, sharply pointed structure, without setae on its tip (Fig. 2a). The basal part of the phallus is clearly visible, pale, oar-like, without setae. It is supported by rather long and thin sclerotized arms (Fig. 2b). The aedeagus is short and oval shaped.

*Anoecia corni* (Fabricius, 1775) (male wingless, dwarfish)—external genitalia are very similar to those of *A*. *vagans*, with less sharply pointed modified parameres (Fig. 2c).

#### Thelaxinae

*Glyphina betulae* (Linne, 1758) (male wingless, dwarfish)—parameres are present, located above the basal part of the phallus, strongly modified and fused into a single, dark, bulb-shaped structure with a few, short setae on its tip (Fig. 2d). The basal part of the phallus is present, but strongly reduced, dusky, triangular, without setae, visible both in the scanning electron microscope (Fig. 2e) and in the slide-mounted specimens (Fig. 2f). Sclerotized arms are barely visible. The aedeagus is rather short with its distal part wider than its basal part (Fig. 2f).

*Glyphina jacutensis* Mordvilko, 1931 (male wingless, dwarfish)—external genitalia are very similar to those of *G*. *betulae* with a barely visible the basal part of the phallus.

#### Eriosomatinae

**Fig. 3:**
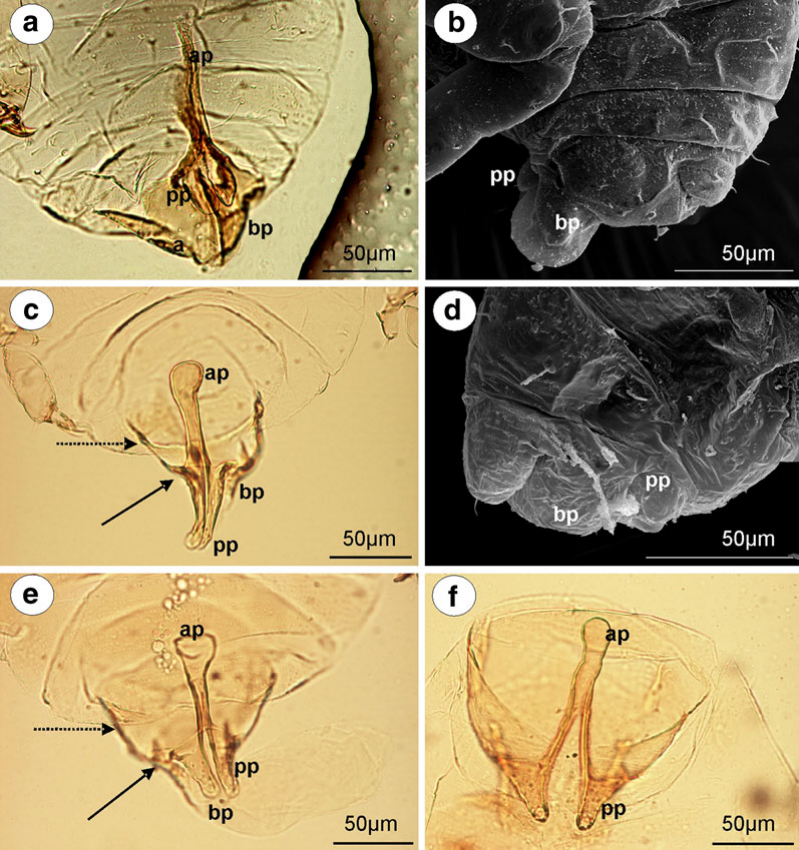
External genitalia of males. **a, b** *Tetraneura (Tetraneurella) nigriabdominalis*. **a** ventral view (light microscopy), **b** lateral view (scanning electron microscopy). **c, d** *Pemphigus versicarius*. **c** ventral view (light microscopy), **d** lateral view (scanning electron microscopy). **e** *Pachypappa tremulae* ventral view (light microscopy). **f** *Prociphilus fraxini* ventral view (light microscopy). *bp* basal part of phallus with sclerotized arms consists of short proximal (*solid arrow*) and long distal (*dotted arrow*) part, *ap* parallel arms of parameres projections, *pp* parameres projections

*Tetraneura (Tetraneurella) nigriabdominalis* (Sasaki, 1899) (male wingless, dwarfish)—parameres are reduced to dusky, lobate projections (Fig. 3a). The basal part of the phallus is dusky, flattened and short, without setae (Fig. 3b). The sclerotized arms are dusky, rather short, of a similar length to the proximal and distal parts. The most significant character of external genitalia, visible in the slide-mounted specimens, is the additional sclerotization of parameres projections reaching up to the VI abdominal sternite. This structure is formed by elongated, parallel arms that are fused together along the entire length (Fig. 3a). The aedeagus is short and oval shaped.

*Pemphigus versicarius* Passerini, 1861 (male wingless, dwarfish)—parameres are reduced to pale, very long finger-like projections. Additional sclerotization of parameres projections is formed by elongated, parallel arms that are fused together along the entire length. They reach up to the VIII abdominal sternite (Fig. 3c). The basal part of the phallus is clearly visible, pale and lobate, without setae (Fig. 3d). The sclerotized arms are dusky, rather short, of a similar length to the proximal and distal parts (Fig. 3c). The aedeagus is not visible.

*Pachypappa tremulae* (Linne, 1761) (male wingless, dwarfish)—external genitalia are similar to those of *Pemphigus versicarius* with much shorter projections of parameres and few short setae on the basal part of the phallus (Fig. 3e).

*Prociphilus fraxini* (Fabricius, 1777) (male wingless, dwarfish)—parameres are reduced to dusky, triangular projections. Additional sclerotization of parameres projections, reaching up to the VIII abdominal sternite, is formed by elongated, separate, parallel arms that are fused together on the tip. The basal part of the phallus is clearly visible, dusky and flattened, without setae. The sclerotized arms are dusky, rather short, of a similar length to the proximal and distal parts (Fig. 3f). The aedeagus is not visible.

*P. ligustrifoliae* (Tseng & Tao, 1938) (male wingless, dwarfish)—external genitalia are similar to those of *Prociphilus fraxini* with finger-like projections of parameres. The aedeagus is short and bulbous.

#### Greenideinae

**Fig. 4:**
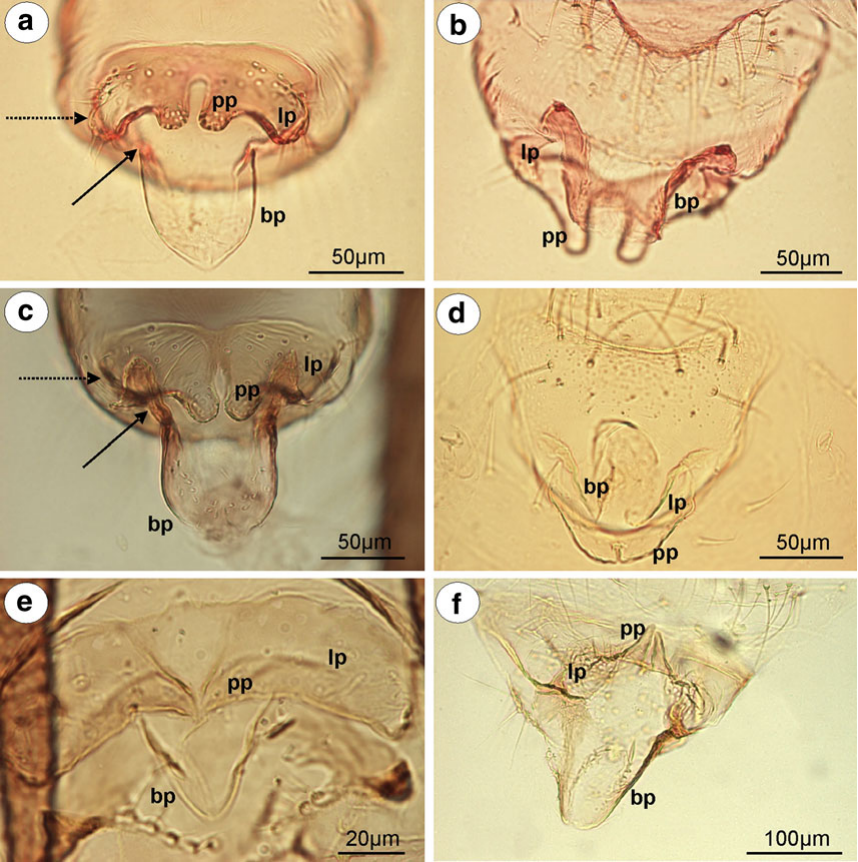
External genitalia of males. **a** *Greenidea (Trichosiphum) carpini* ventral view. **b** *Eutrichosiphum sinense* dorsal view. **c** *Mollitrochosiphum (Metatrichosiphon) yamabiwae* ventral view. **d** *Anomalosiphum mendeli* ventral view. **e** *Schoutedenia ralumensis* ventral view. **f** *Aiceona japonica* ventral view (light microscopy). *bp* basal part of phallus with sclerotized arms consists of short proximal (*solid arrow*) and long distal (*dotted arrow*) part, *lp* lobate parts of parameres, *pp* parameres projections

*Greenidea (Trichosiphum) carpini* Takahashi, 1963 (male winged)—parameres are present, located above the basal part of the phallus, pale, clearly divided into lobate parts which arising into short, finger-like projections toward base of the phallus. Lobate parts of parameres are basally fused. The outer margin of lobate parts of parameres is covered by numerous, long setae, whereas on projections, setae are short and distributed on their apices. The basal part of the phallus is rather long, pale, sclerotized on the outer margins, without setae. The sclerotized arms are dusky, rather short, of a similar length to the proximal and distal parts (Fig. 4a). The aedeagus is not visible.

*G. (T.) okajimai* Suenaga, 1943 (male winged)—external genitalia are similar to those of *Greenidea (Trichosiphum) carpinii* with wider and much more setose lobate parts of parameres and wider the basal part of the phallus.

*Eutrichosiphum sinense* D. N. Raychaudhuri, 1956 (male winged)—parameres are present, clearly visible, pale, basally fused, free at their finger-like projections. Lobate parts are setose in comparison with their projections. The basal part of the phallus is rather short, pale, without setae, fused, together forming an almost complete cylinder. The sclerotized arms are dusky, rather short, of a similar length to the proximal and distal parts (Fig. 4b). The aedeagus is not visible.

*Mollitrochosiphum (Metatrichosiphon) yamabiwae* Suenaga, 1943 (male winged)—parameres are present, clearly visible, dusky, basally fused, free at their finger-like projections. In comparison with the above-mentioned species, both the lobate parts of parameres and their projections are setose and much more robust. The basal part of the phallus is long, flattened, hook-shaped, pale, without setae. The sclerotized arms are dusky, rather short, of a similar length to the proximal and distal parts (Fig. 4c). The aedeagus is not visible.

*Anomalosiphum mendeli* Quednau & Martin 2006 (male winged)—parameres are present, clearly visible, pale, basally fused, free only at their very short, finger-like projections. They are almost hairless, having only a few short setae on the outer margin of the lobate parts of parameres. The basal part of the phallus is rather short and flattened, pale, without setae. The sclerotized arms are rather short, of a similar length to the proximal and distal parts (Fig. 4d). The aedeagus is not visible.

*Schoutedenia ralumensis* Rübsaamen, 1905 (male winged)—parameres are present, clearly visible, basally fused, pale, with the lobate parts arising into sharply pointed projections toward the base of the phallus. The projection is short and hairless in comparison with the setose lobate part of parameres. The basal part of the phallus is short, hook-shaped, pale, without setae. The sclerotized arms are barely visible (Fig. 4e). The aedeagus is not visible.

#### Aiceoninae

*Aiceona japonica* Takahashi, 1960 (male winged)—parameres are present, located above the basal part of the phallus, clearly visible, separate, pale, setose, with the lobate parts arising into sharply pointed projections toward the base of the phallus. The projection is short and hairless. The basal part of the phallus is rather long, pale, hook-shaped, without setae. The sclerotized arms are clearly visible and dusky with a long distal part and a short proximal part (Fig. 4f). The aedeagus is not visible.

#### Lachninae

**Fig. 5:**
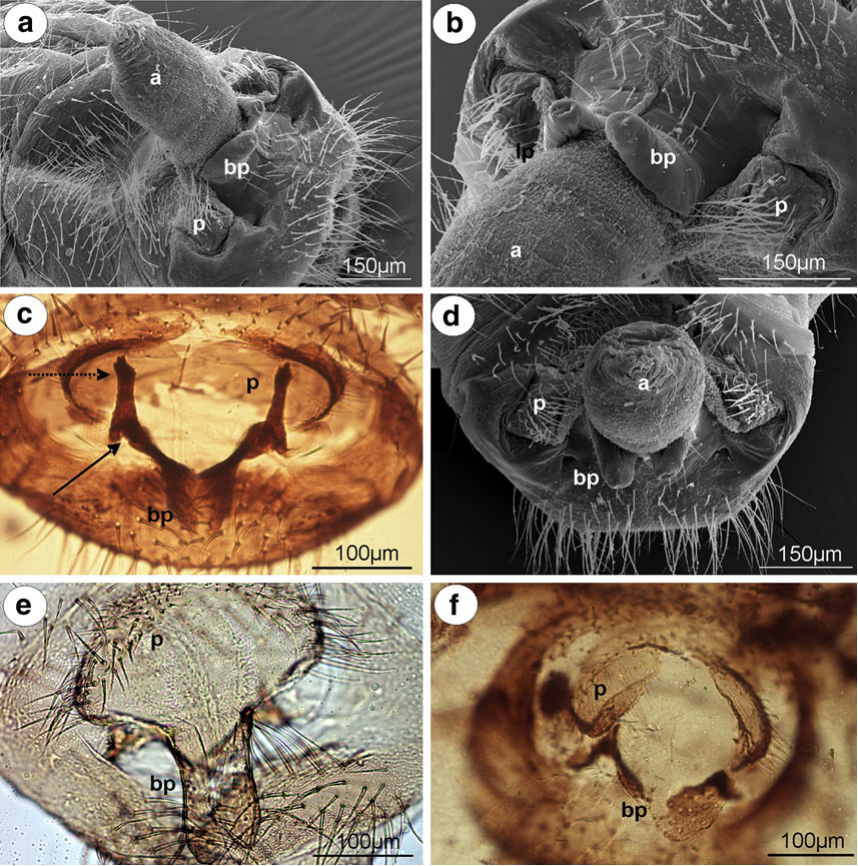
External genitalia of males. **a, b,** **c** *Lachnus pallipes*. **a** lateral view (scanning electron microscopy), **b** dorsal view (scanning electron microscopy), **c** ventral view (light microscopy). **d, e** *Lachnus roboris*. **d** ventral view (scanning electron microscopy), **e** ventral view (light microscopy). **f** *Pterochloroides persicae* ventral view (light microscopy). *a* aedeagus, *bp* basal part of phallus with sclerotized arms consists of short proximal (*solid arrow*) and long distal (*dotted arrow*) part, *p* parameres

*Lachnus pallipes* (Hartig, 1841) (male wingless)—parameres are present, clearly visible, separate, semicircular, dusky on the outer margin, with numerous, long setae mostly distributed on the outer margin (Fig. 5a). In comparison with the above-mentioned species, parameres are positioned laterally to the basal part of the phallus (Fig. 5b). The basal part of the phallus is short, finger-like, dark pigmented, with only a few rather long setae (Fig. 5a). The sclerotized arms are of a similar length to the proximal and distal parts, robust and clearly visible. Distal part ends in forked apices (Fig. 5c). The aedeagus is short and bulbous (Fig. 5a).

*Lachnus roboris* (Linne, 1758) (male winged)—parameres are present, located laterally to the basal part of the phallus, clearly visible, separate, semicircular, pale, with numerous, long setae on the entire surface (Fig. 5d). The basal part of the phallus is wide, pale, without setae (Fig. 5e). Sclerotized arms are similar to those of the above-mentioned species but much wider. The aedeagus is short and bulbous (Fig. 5d).

*Pterochloroides persicae* (Cholodkovsky, 1899) (male winged)—parameres are present, located above the basal part of the phallus, clearly visible, separate, lobe-shaped, dusky on the outer margin, with numerous, rather short setae on the entire surface. The basal part of the phallus is short, flattened, oval paddle-shaped, dusky, with setae. The sclerotized arms are clearly visible, very short, of a similar length to the proximal and distal parts (Fig. 5f). The adeagus is not visible.

**Fig. 6:**
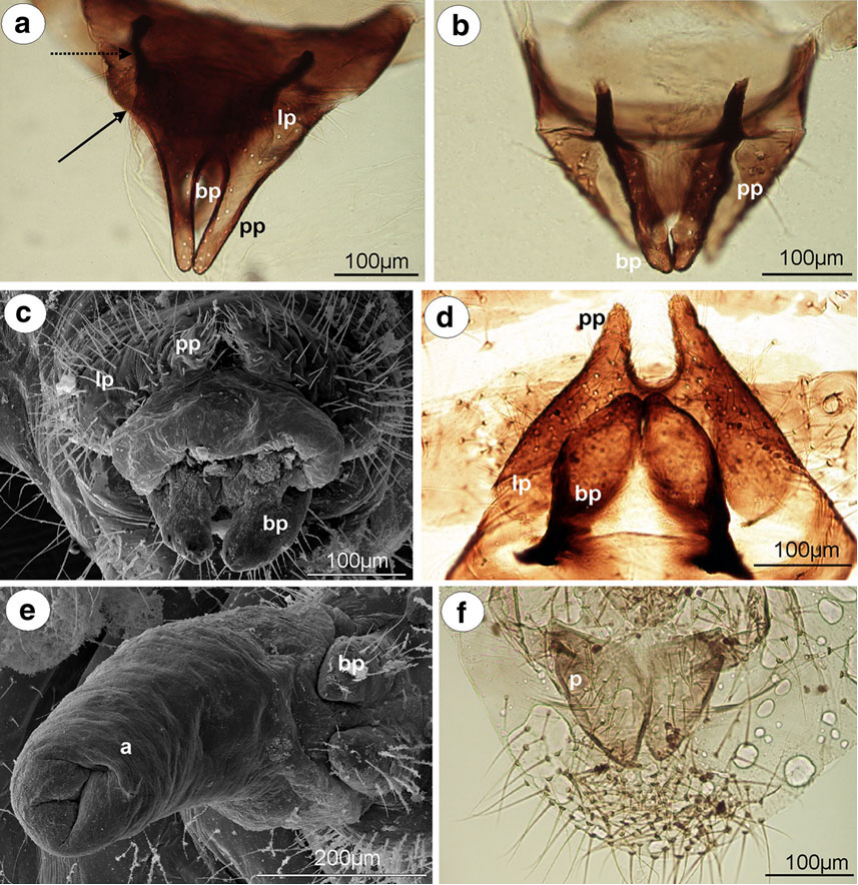
External genitalia of males. **a** *Stomaphis aceris* ventral view (light microscopy). **b** *S. pini* dorsal view (light microscopy). **c, d, e,** **f** *Stomaphis quercus*. **c** ventral view (scanning electron microscopy), **d** ventral view (light microscopy), **e** lateral view (scanning electron microscopy), **f** nymph ventral view (light microscopy). *a* aedeagus, *bp* basal part of phallus with sclerotized arms consists of short proximal (*solid arrow*) and long distal (*dotted arrow*) part, *p* parameres, *lp* lobate parts of parameres, *pp* parameres projections

*Stomaphis aceris* Takahashi, 1960 (male wingless, dwarfish)—parameres are present, located above the basal part of the phallus, clearly visible, separate, pale, divided into lobate parts arising into a finger-like projection toward the base of the phallus. The projection is very thin and long (the longest among the species of the genus *Stomaphis* studied and twice as long as the basal part of the phallus) and almost hairless in comparison with the lobate part of paramere. The basal part of the phallus is short, finger-like, dark pigmented, sclerotized on inner margin, slightly setose. The sclerotized arms are clearly visible, of a similar length to the proximal and distal parts, both very short, dark pigmented (Fig. 6a). The adeagus is not visible.

*Stomaphis graffi* Cholodkovsky, 1894 (male wingless, dwarfish)—external genitalia are similar to those of the above-mentioned species with a short and thin projection (2 times shorter than the basal part of the phallus). The basal part of the phallus is long, finger-like, setose only on apices. The aedeagus is not visible. Nymphs with clearly visible, broadly rounded buds of parameres situated in the genital area.

*Stomaphis pini* Takahashi, 1920 (male wingless, dwarfish)—projections of parameres are very short, wide, setose, as long as the basal part of the phallus. The basal part of the phallus is thin and flattened, dark pigmented, sclerotized on outer margin and slightly setose. The sclerotized arms are rather short, of a similar length to the proximal and distal parts (Fig. 6b). The aedeagus is not visible.

*Stomaphis quercus* (Linne, 1758) (male wingless, dwarfish)—projections of parameres are finger-like, rather short and wide, covered by much shorter setae than the lobate part of paramere (Fig. 6c). The basal part of the phallus is rather short, flattened, club-shaped, dusky, sclerotized on inner margin, with numerous, long setae (Fig. 6c). The sclerotized arms are dark pigmented and much more robust than in the above-mentioned species (Fig. 6d). The aedeagus is short (Fig. 6e). Nymphs with clearly visible, broadly rounded buds of parameres situated in the genital area (Fig. 6f).

*Stomaphis yanonis* Takahashi, 1918 (male wingless, dwarfish)—external genitalia are similar to those of *S. aceris* with shorter, paler and much more setose projections of parameres.

**Fig. 7:**
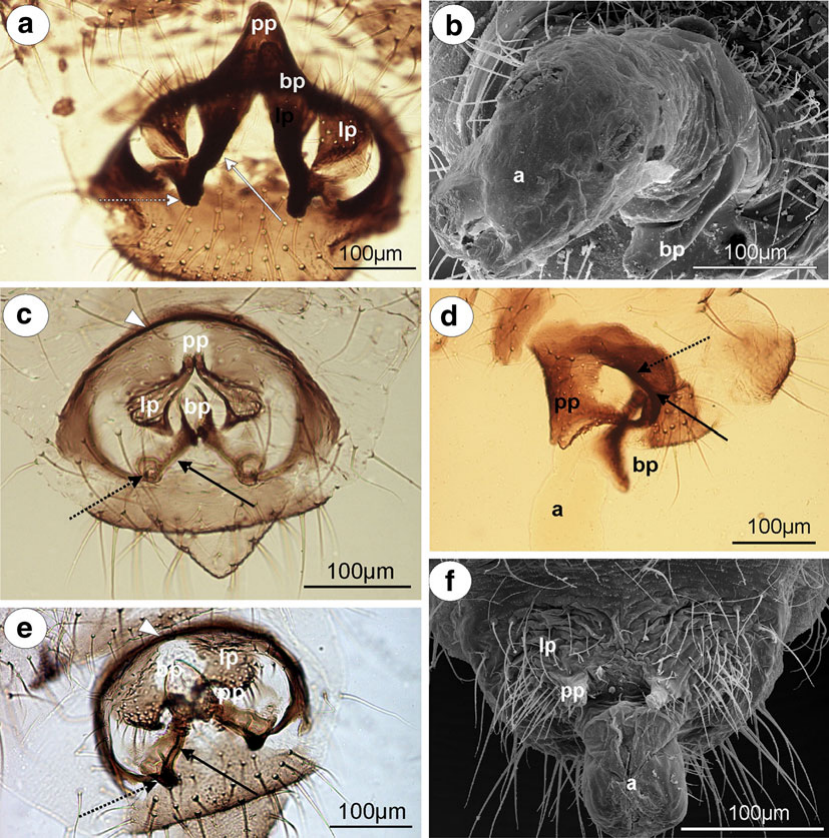
External genitalia of males. **a, b** *Maculolachnus submacula* **a** ventral view (light microscopy), **b** lateral view (scanning electron microscopy). **c** *Schizolachnus piniradiatae* ventral view (light microscopy), **d, e** *Eulachnus agilis* **d** lateral view, **e** ventral view (light microscopy). **f** *Eulachnus rileyi* ventral view (scanning electron microscopy). *a* aedeagus, *bp* basal part of phallus with sclerotized arms consists of short proximal (*solid arrow*), long distal (*dotted arrow*) part and upper half-circle-shaped structure that surrounds the genital area (*arrow*-*head*), *lp* lobate parts of parameres, *pp* parameres projections

*Maculolachnus submacula* (Walker, 1848) (male wingless)—parameres are present, located above the basal part of the phallus, clearly visible, dusky, with separate, setose lobate parts and their projections fused into a single, triangular structure. The basal part of the phallus is long, robust, paddle-shaped, dusky, with a few short setae. The distal part of sclerotized arms is rather short and thin, and proximal part is robust, strongly sclerotized, and dark pigmented (Fig. 7a). The aedeagus is short and bulbous (Fig. 7b).

*Schizolachnus piniradiatae* (Davidson, 1909) (male winged)—parameres are present, located above the basal part of the phallus, clearly visible, basally separate, divided into lobate parts arising into long, finger-like projection toward the base of the phallus. The parameres are dusky, with numerous, long setae on the entire surface. The basal part of the phallus is short, flattened, dusky, and setose. The sclerotized arms are clearly visible, dark pigmented with proximal part robust and ends in rounded apices. The distal part is thinner and forms the upper half-circle-shaped structure that surrounds the genital area (Fig. 7c). The aedeagus is not visible.

*Eulachnus agilis* (Kaltenbach, 1843) (male winged)—parameres are present, located above the basal part of the phallus, clearly visible, basally fused. Their lobate parts (triangular in lateral view Fig. 7d) arise into a distinct, finger-like projection toward the base of the phallus. The parameres are dark pigmented on the outer margin, with numerous, long setae on the entire surface with the exception of a few, short setae distributed on the projections. The basal part of the phallus is short, flattened, pale, with a few short setae. The sclerotized arms are clearly visible, strongly sclerotized, and dark pigmented. The proximal part is robust and ends in oval apex. The distal part is thinner and forms the upper half-circle-shaped structure that surrounds the genital area (Fig. 7e). The aedeagus is not visible.

*Eulachnus rileyi* (Williams, 1911) (male winged)—external genitalia are similar to those of *E. agilis* with a thinner basal part of the phallus. The aedeagus is short and bulbous (Fig. 7f).

**Fig. 8:**
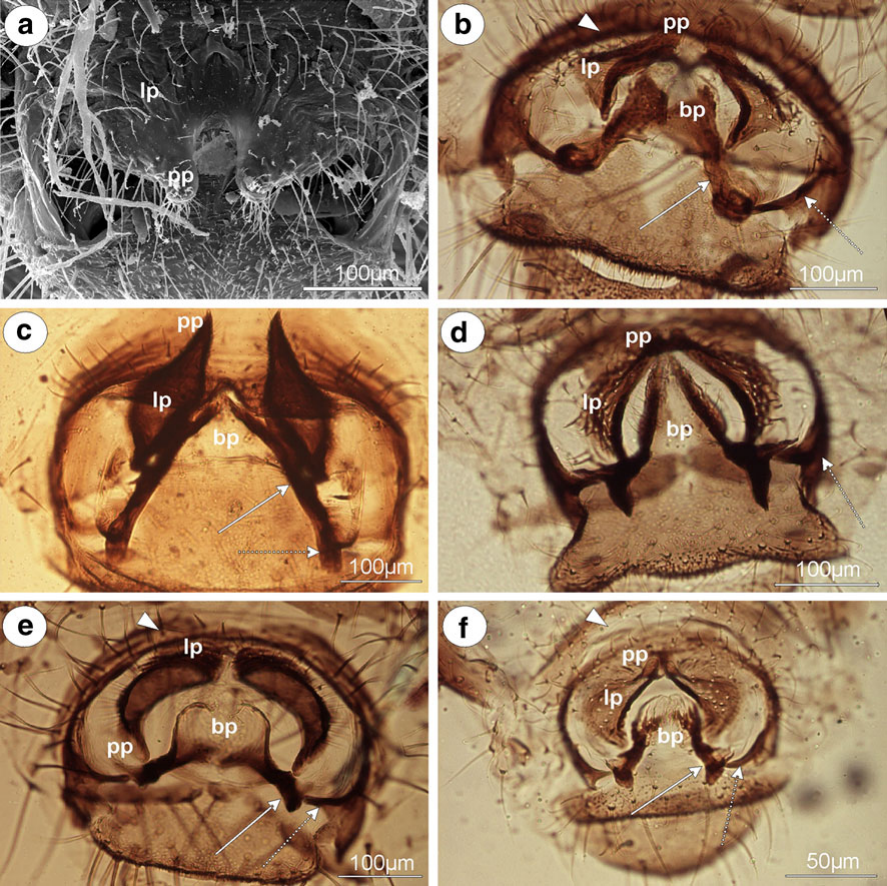
External genitalia of males. **a, b** *Cinara cedri* **a** ventral view (scanning electron microscopy), **b** dorsal view (light microscopy). **c** *Cinara maculipes* ventral view (light microscopy). **d** *Cinara piceicola* ventral view (light microscopy). **e** *Cinara todocola* ventral view (light microscopy). **f** *Cinara (Cedrobium) laportei* ventral view (light microscopy). *bp* basal part of phallus with sclerotized arms consists of short proximal (*solid arrow*), long distal (*dotted arrow*) part and upper half-circle-shaped structure that surrounds the genital area (*arrow*-*head*), *lp* lobate parts of parameres, *pp* parameres projections

*Cinara cedri* Mimeur, 1936 (male winged)—parameres are present, located above the basal part of the phallus, clearly visible, dark pigmented, with setose lobate parts fused and arising into a finger-like projections toward base of the phallus (Fig. 8a). The projection is long (twice as long as the basal part of the phallus) and covered with numerous, long setae. The basal part of the phallus is rather short, flattened, dark pigmented, and slightly setose. The sclerotized arms are clearly visible, strongly sclerotized, and dark pigmented. The proximal part is robust and ends in oval apex. The distal part is thinner and forms the upper half-circle-shaped structure that surrounds the genital area (Fig. 8b). The aedeagus is not visible.

*Cinara laricis* (Hartig, 1839) (male winged)—external genitalia are similar to those of *C. cedri* with shorter and paler projections of parameres.

*Cinara maculipes* Hille Ris Lambers 1966 (male winged)—projections of parameres are sharply pointed, triangular, setose, dark pigmented (Fig. 8c). The basal part of the phallus is slightly visible, in lateral view almost rounded with more sclerotized outer margin. The aedeagus is not visible.

*Cinara piceicola* (Cholodkovsky, 1896) (male wingless)—lobate parts of parameres are broadly rounded, setose, and the projections of parameres are very long, finger-like, setose and dark pigmented. The basal part of the phallus is clearly visible, very thin and long, almost parallel, dark pigmented, setose. The sclerotized arms are clearly visible. The proximal part is robust, dark pigmented and ends in spiniform apices. The distal part is much thinner and forms the upper half-circle-shaped structure that surrounds the genital area (Fig. 8d). The aedeagus is not visible.

*Cinara todocola* (Inouye, 1936) (male winged)—lobate parts of parameres are crescent-shaped, and the projections of parameres are short and sharply pointed, dark pigmented, setose on the entire surface. The basal part of the phallus is hook-shaped, short, pale, without setae. The proximal part of sclerotized arms is robust, dark pigmented and ends in a spiniform apices. The distal part is much thinner and forms the upper half-circle-shaped structure that surrounds the genital area. (Fig. 8e). The aedeagus is not visible.

*Cinara (Cedrobium) laportei* (Remaudiere, 1954) (male winged)—external genitalia are similar to those of *C. cedri* with slight projections of parameres and less robust sclerotized arms (Fig. 8f).

## Discussion

The previous study of the comparative morphology of the external male genitalia of Aphididae (Wieczorek et al. 2011) showed that these structures are well developed and typically consist of the phallus, which is composed of the sclerotized basal part with its articulation and a membranous apical part, the aedeagus. Above the phallus, there is a pair of setose parameres. However, this scheme was characteristic mainly for taxa of a normal size (i.e., not dwarfish) and mostly for winged males. The present study shows that in almost all of the species examined (representatives of eight taxa traditionally classified as subfamilies within 24 subfamilies of the Aphididae), the external male genitalia are modified in both dwarfish and normal-sized males (winged and wingless).

### The external genitalia of males—the phallus

In Aphididae, the phallus is composed of a membranous part—the aedeagus and the sclerotized basal part supported by its articulations. The aedeagus is entirely unsclerotized and its shape is estimated mostly based on material preserved in alcohol or with scanning electron microscope. In most of the species studied, it is clearly visible, although it is rather short and oval shaped (*Lachnus pallipes* Fig. 5a and *Stomaphis quercus* Fig. 6e). Only in *Glyphina betulae* is the distal part of the aedeagus bulbous and wider than its proximal part (Fig. 2f). Polaszek (1987b in lit.), according to the terminology of Bonhag and Wick (1953), distinguished the basal part of the aedeagus—a conjuctiva and its distal part—a vesica in some species. The present study does not support this division. Generally, in the species studied, the aedeagus (when visible) is characterized by a small amount of variation in its shape in comparison with previously studied species of Drepanosiphinae, Calaphidinae, Chaitophorinae or Aphidinae.

The basal part of the phallus (the so-called penis valves by some authors), which is composed of the partially sclerotized lobes, is present and clearly visible in all of the species studied. Only in the dwarfish males of *Glyphina betulae* is it reduced to small, triangular plates (Fig. 2e). Polaszek (1987b in lit.) stated that in representatives of the genera *Anoecia*, *Glyphina*, *Thelaxes,* and *Pemphigus* (all with dwarfish males), the basal part of the phallus is either absent, modified and fused or is replaced by an extension of the anal plate. The present study does not support this observation. In all of the species with dwarfish males studied, representatives of the Mindarinae, Hormaphidinae, Anoeciinae, Thelaxinae and Eriosomatinae as well as dwarf males of the genus *Stomaphis* (Lachninae), the basal part of the phallus is present and visible in the scanning electron microscope and the slide-mounted specimens. In most cases, the lobes are rather short and flattened (*Nipponaphis distyliicola* Fig. 1e, *Pemphigus versicarius* Fig. 3d), forceps-like (*Neothoracaphis yanonis* Fig. 1d, *Monzenia globuli* Fig. 1f), triangular (*Mindarus abietinus* Fig. 1a, *Anoecia vagans* Fig. 2b) or finger-like (*Stomaphis aceris* Fig. 6a). Representatives of the Greenideinae, Aiceoninae, and Lachninae (in exception of the genus *Stomaphis*) are characterized by normal-sized males. In most of species of the Greenideinae studied, the basal part of the phallus is rather short and flattened (*Anomalosiphum mendeli* Fig. 4d) or hook-shaped (*Mollitrochosiphum (Metatrichosiphon) yamabiwae* Fig. 4c), usually without setae. However, the shape of basal part of the phallus in *Eutrichosiphum sinense* differs from these structures in other aphid genera. The lobes are fused, free only at their tips and together form an almost complete cylinder (Fig. 4b). Unfortunately, only slide-mounted males were available for Greenideinae so it was not possible to perform a detailed electron microscopy study of these structures. The basal part of the phallus of *Aiceona japonica* is similar to the hook-shape of the above-mentioned species of Greenideinae; however, it is much longer (Fig. 4f). Lachninae are characterized by the most various shapes of the basal part of the phallus. In general, the most common is the finger-like shape (*Lachnus roboris* Fig. 5e, *Cinara piceicola* Fig. 8d) and rarely club-shaped (*Stomaphis quercus* Fig. 6d) or paddle-shaped (*Maculolachnus submacula* Fig. 7a). It is joined laterally with the aedeagus and attached to it (Figs. 5b, 6e). Moreover, the basal part of the phallus is more setose in Lachninae than in representatives of the other subfamilies.

In all of the species studied, the basal part of the phallus is additionally fortified by sclerotized arms. These structures are not uniform and vary in form and length. In representatives of the Hormaphidinae, Anoeciinae (*Anoecia vagans* Fig. 2b), Thelaxinae, Eriosomatinae (*Pemphigus versicarius* Fig. 3c), and Greenideinae (*Mollitrochosiphum (Metatrichosiphon) yamabiwae* Fig. 4c), the sclerotized arms are barely visible, pale, rather short and of a similar length to the proximal and distal parts. In Mindarinae (*Mindarus japonicus* Fig. 1c) and Aiceoninae, the sclerotized arms are clearly visible and dusky with a long distal part and a short proximal part. In Lachninae, the sclerotized arms are the most variable: of a similar length to the proximal and distal parts in the genera *Lachnus* (Fig. 5c), *Pterochloroides* and *Stomaphis* (Fig. 6a) with a rather short and thin distal part and a robust proximal part in *Maculolachnus submacula* (Fig. 7a), or with a robust proximal part ending in an oval apices and a much thinner distal part, which forms the upper half-circle-shaped structure that surrounds the genital area in the genera *Schizolachnus* (Fig. 7c), *Eulachnus* (Fig. 7e) and *Cinara* (Fig. 8b–f). Additionally, in all of the species of this subfamily studied, the arms are heavily sclerotized, robust and dark pigmented.

### The external genitalia of males—parameres

Parameres are the most variable part of the male external genitalia of species studied. In *Mindarus* (Fig. 1a), which has dwarfish males, parameres are rather small, fused basally and elongated, similar to those of previously studied species of Drepanosiphinae, Calaphidinae, Chaitophorinae, or Aphidinae. The dwarfish males of Hormaphidinae (*Neothoracaphis yanonis* Fig. 1d) have a similar type of broadly rounded and barely visible parameres. On the other hand, parameres appear to be significantly modified in the dwarfish males of the Anoeciinae, Thelaxinae, and Eriosomatinae. In representatives of the genera *Anoecia* and *Glyphina*, parameres are fused into a single, sharply pointed structure that is triangular without setae on the tip in *Anoecia* (Fig. 2a–c) or bulb-shaped with few, short setae on the tip in *Glyphina* (Fig. 2d). In the species of Eriosomatinae studied, parameres are reduced to variable-shaped projections—mostly finger-like (*Pemphigus versicarius* Fig. 3c) but also lobate (*Tetraneura (Tetraneurella) nigriabdominalis* Fig. 3a) or triangular (*Prociphilus fraxini* Fig. 3f)—and are supported by additional sclerotization that reaches up to the VI-VIII abdominal sternite. This structure has never been described before and is formed by elongated, parallel arms fused together along the entire length (Fig. 3a) or fused only on the tip (Fig. 3f). Only de Fluiter (1931) called it a “ventrale chitinestaaf” (a chitinized rod) and figured it in *Eriosoma lanigerum*. As in a case of the sclerotized arms of basal part of the phallus, they are probably apophyses that provide support for muscle attachment. In male insects, parameres are the grasping apparatus, which play an important role during copulation. It is possible that the modified parameres of Eriosomatinae need additional support; however, the mating behavior of these aphids has never been observed. Greenideinae are also characterized by modified parameres. They are clearly divided into lobate parts arise into short, usually finger-like projections toward the base of the phallus. The lobate parts of parameres are fused basally and are free only at their projections. Polaszek (1987b in lit.) stated that parameres of Greenideinae are reduced and only the structure he called the anterior genital process, which forms a pair of finger-like processes, is developed. The present study does not support this observation. All of the species studied, especially representatives of the tribe Greneeidini (Fig. 4a–c), have large, well-developed parameres that are clearly visible in the slide-mounted specimens. Chaudhuri (1956) and Chakrabarti and Maity (1980) also described and illustrated parameres of Greneeidini as composed of the base with lateral projections. Moreover, scanning electron micrographs of the external genitalia of males of *Schoutedenia ralumensis* presented by Polaszek (1987b in lit.) show that these structures are well developed. *Aiceona japonica* (Aiceoninae) has a similar type of parameres as those found in Greenideinae (Fig. 4f). All of the mentioned species have parameres positioned above the basal part of the phallus. The most diverse forms of parameres are characterized for Lachninae. They are separate, semicircular or lobate without a finger-like projection in the genera *Lachnus* (Fig. 5c, e) and *Pterochloroides* (Fig. 5f) (Lachnini) or divided into lobate parts (separate or fused) arising into variable-shaped projections toward the base of the phallus in the remaining species of this subfamily that were studied. The projections of the lobate parts of parameres (called the anterior genital process by Polaszek 1987b in lit.) are variable between different genera and species, but they vary very little within each species. Their shape and length is a very good diagnostic character for distinguishing males belonging to the different species of this subfamily. Those observed in the genera *Stomaphis* (Lachnini—Fig. 6a, b, d) and *Cinara* (Eulachnini—Fig. 8b–f) are the most variable. In *Maculolachnus submacula* (Lachnini—Fig. 7a), the projections of the lobate parts of parameres are fused into a single, large, triangular structure. Moreover, nymphs of the genus *Stomaphis* have clearly visible, broadly rounded buds of parameres situated in the genital area (Fig. 6f). Parameres of Lachninae are more setose than in the other subfamilies. In the genus *Lachnus* they are positioned laterally to the basal part of the phallus (*Lachnus pallipes* Fig. 5b), whereas in the remaining species of this subfamily, parameres are located above the basal part of the phallus.

### The external genitalia of males—phylogenetic implications

Comparative studies of male genitalia have formed the basis of the taxonomic and phylogenetic studies of many groups of insects (e.g., Zumpt and Heinz 1950; Eades 2000). Males of most Aphididae, unlike males of other Hemiptera, do not appear until autumn and have to mature quickly. This is why the taxonomy and construction of the higher classifications of this group of insects have generally been based on the morphology and the characters of the females. However, a comparative, systematic study of the male genitalia of Aphididae has revealed a number of characters that are of potential use in discussions of the phylogenetic relationships of this group of Hemiptera.

#### Phylogenetic implications—why the phallus presents some stable characters.

The phallus, which is composed of the sclerotized basal part with its articulation and a membranous apical part—the aedeagus, presents some stable characters among all of the subfamilies studied, that is, with dwarfish, normal-sized, winged or wingless males. The stable character of the components of the phallus is a result of their functions: the lateral sclerotization of the otherwise membranous aedeagus enfolds it and supported by the sclerotized arms takes part in everting the aedeagus and maintaining it in the right position during copulation. The shape and size of the aedeagus and the basal part of the phallus as well as the length of the sclerotized arms is variable between different genera and species, but varies very little within each species. Thus, this characteristic is valuable for diagnosing species but is less important in phylogenetic considerations.

#### Phylogenetic implications—what is important parameres not modified or modified?

**Fig. 9:**
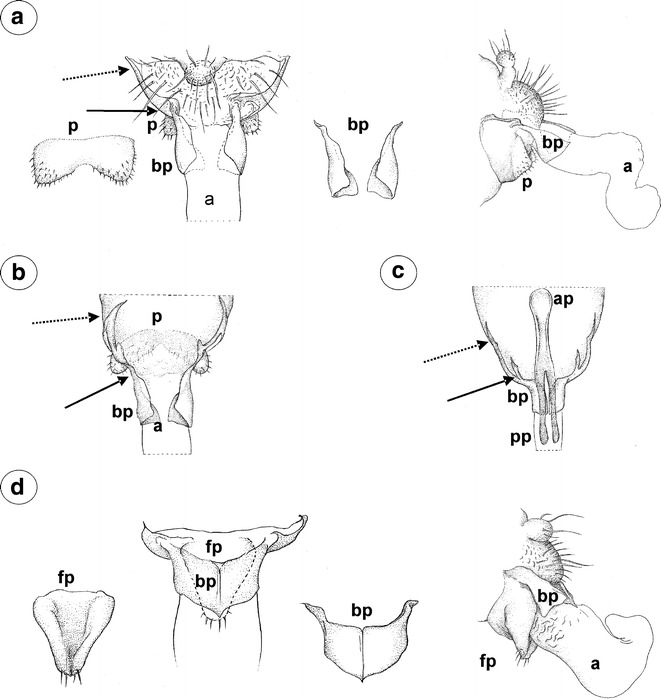
External genitalia of males. **a** Not modified in the Drepanosiphinae, Chaitophorinae, Calaphidinae, Phyllaphidinae, Saltusaphidinae, Lizeriinae, Spicaphidinae, Tamalinae, Parachaitophorinae, Phloeomyzinae and Aphidinae. **b** Not modified in the Mindarinae and Hormaphidinae. **c** Modified with parameres reduced to variable-shaped projections in the Eriosmatinae. **d** Modified with parameres fused into a single, sharply pointed structure in the Anoeciinae and Thelaxinae. *a* aedeagus, *bp* basal part of phallus with sclerotized arms consists of short proximal (*solid arrow*) and long distal (*dotted arrow*) part, *ap* parallel arms of parameres projections, *fp* fused parameres, *p* parameres, *pp* parameres projections. Drawings according to light microscopical preparations

On the other hand, parameres show the most striking differences and appear to be highly variable among the different subfamilies studied. In Drepanosiphinae, Chaitophorinae, Calaphidinae, Phyllaphidinae, Saltusaphidinae, Lizeriinae, Spicaphidinae, Tamalinae, Parachaitophorinae, Phloeomyzinae, and Aphidinae (Fig. 9a), parameres are large and positioned at the anterior end of the genital area, above the basal part of the phallus (Wieczorek et al. 2011). Similar types of parameres have been found in the dwarfish males of the Mindarinae (Fig. 9b) and Hormaphidinae. The Mindarinae, represented by only one extant genus *Mindarus* Koch, are considered to be the most primeval drepanosiphine aphids by some authors (Takahashi 1931; Mordvilko 1934; Heie and Węgierek 2009b; Quednau 2010) or are recognized as an independent subfamily (Heie 1980; Remaudiere and Stroyan 1984). In comparison with other taxa with dwarfish males, parameres of *M*. *abietinus* and *M. japonicus* are not modified and are similar to those observed in drepanosiphine aphids. Thus, our study corroborates Quednau’s (2010) point of view, who claims that this genus represents the most primordial group of the drepanosiphine aphids. The dwarfish males of Hormaphidinae that were studied represent the same type of parameres, although they are very small. According to Quednau (2010), the Hormaphidinae together with Eriosomatinae and Anoeciinae form the common lineage. However, parameres of studied species belonging to these subfamilies differ significantly. In Eriosomatinae, parameres are reduced to rather long projections, which are supported by additional sclerotization reaching up to the VI–VIII abdominal sternite (Fig. 9c). This unusual shape of parameres may be regarded as the autapomorphy of this subfamily. In Anoeciinae, on the other hand, parameres are fused into a single, sharply pointed structure, similar to that in Thelaxinae (Fig. 9d). It is not clear whether this forward-pointing structure at the anterior end of the genital area actually represents the parameres that have become fused, or whether it is derived from the fusion of parameres’ projections or whether it has arisen in some other way. According to the molecular studies of Ortiz-Rivas and Martinez-Torres (2010), Anoeciinae, Eriosomatinae, Hormaphidinae, Mindarinae, and Thelaxinae are grouped together. Moreover, these subfamilies share a common feature—the presence of a wingless, dwarfish sexual generation (feeding—Anoeciinae, Mindarinae, Thelaxinae, Hormaphidinae or non-feeding—Eriosomatinae). However, they do not share a common origin of host alternation (dioecious—pemphigid host alternation—return of sexuparae to the primary host in Anoeciinae, Eriosomatinae, Hormaphidinae, and monoecious in Mindarinae and Thelaxinae). Von Dohlen and Moran (2000) stated that host alternation originated independently in the ancestors of these aphids. It is probable that the modifications in parameres also arose independently. Moreover, reduction in the size of sexual morphs may then have a direct effect on characters associated with external genitalia, that is, very small and barely visible parameres in Hormaphidinae and very small the basal part of the phallus in Thelaxinae.

**Fig. 10:**
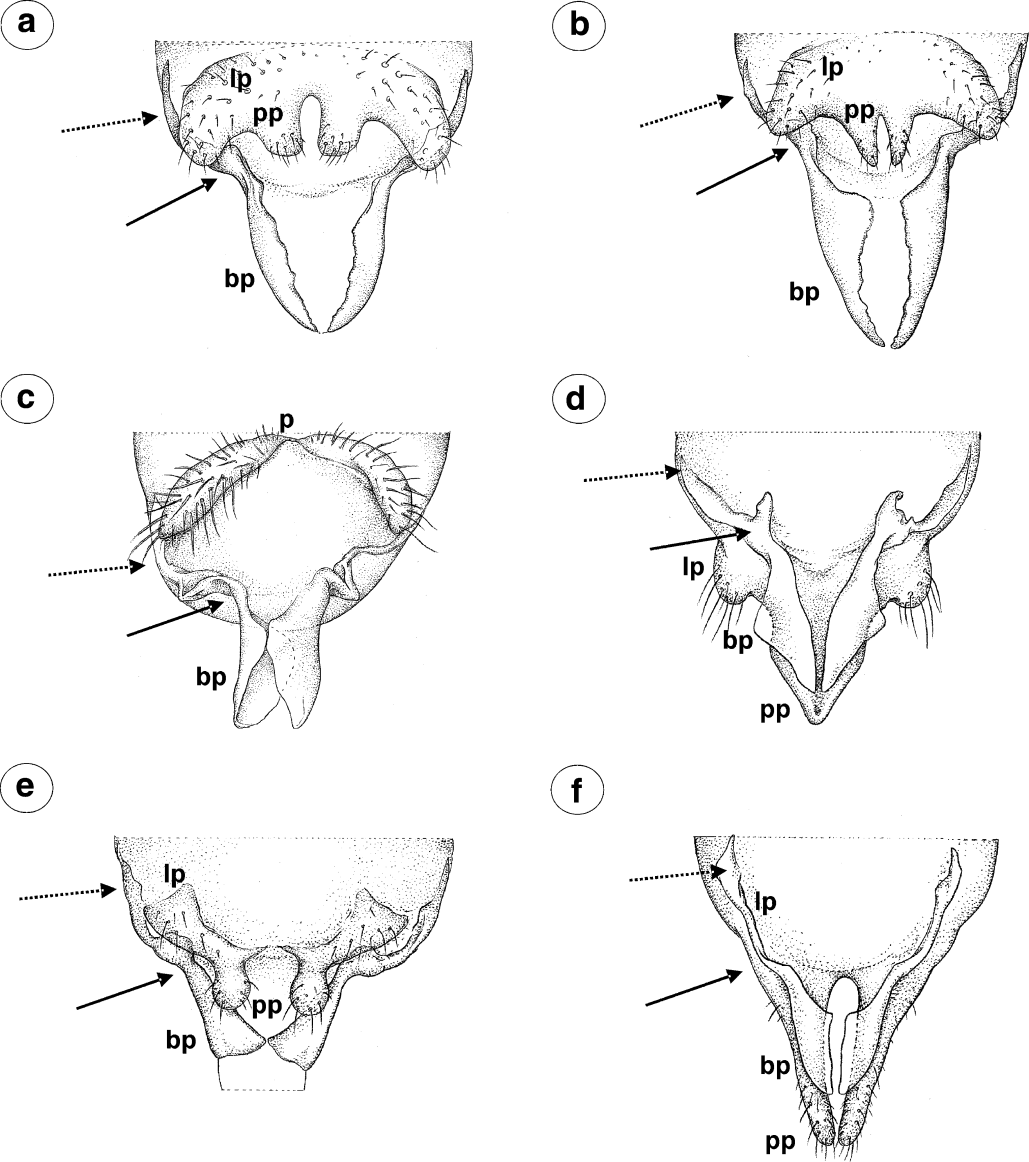
External genitalia of males. **a** Modified with parameres divided into lobate parts arising into finger-like projections in the Greenideinae. **b** Modified with parameres divided into lobate parts arising into finger-like projections in the Aiceoninae. **c** Not modified in genera *Lachnus* and *Pterochloroides* (Lachninae). **d** Modified in the genus *Maculolachnus submacula* (Lachninae) with parameres projections fused into single structure. **e** Modified in the genera *Cinara*, *Eulachnus*, *Schizolachnus* and **f** *Stomaphis* (Lachninae) with parameres divided into lobate parts arising into finger-like projections. *bp* basal part of phallus with sclerotized arms consists of short proximal (*solid arrow*) and long distal (*dotted arrow*) part, *lp* lobate parts of parameres, *p* parameres, *pp* parameres projections. Drawings according to light microscopical preparations

Greenideinae (Fig. 10a) and Aiceoninae (Fig. 10b) are characterized by normal-sized males and large parameres divided into lobate parts arising into short, usually finger-like projections toward the base of the phallus. The close relations between these subfamilies were pointed out by Heie and Węgierek (2009b); however, they remain unsampled for molecular data (von Dohlen 2009). According to Quednau and Martin (2006), the genus *Aiceona* Takahashi may have evolved from *Neophyllaphis*-like ancestors, which may also be the ancestors of primitive Lachninae. In fact, parameres of *A. japonica* are also very similar to those of some *Cinara* species.

The most diverse forms of parameres, similar to almost all of the types described above, are characterized for Lachninae. Parameres of the genera *Lachnus* (Fig. 10c) and *Pterochloroides* resemble those found in Aphidinae and drepanosiphine aphids in shape and size; however, in the genus *Lachnus,* they are positioned laterally to the basal part of the phallus. In *Maculolachnus submacula* (Fig. 10d), projections fused into a single structure, resemble those found in Anoeciinae and Thelaxinae, but the lobate parts of parameres are large and well developed. In the genera *Cinara* (Fig. 10e), *Eulachnus*, *Schizolachnus,* and *Stomaphis* (Fig. 10f) (and in representatives of the tribe Tramini *Trama troglodytes* von Heyden not studied here (Blackman et al. 2001)), parameres divided into lobate parts arising into projections, resemble those found in Greenideinae and Aiceoninae; however, they are highly diverse in the shape and size of their projections. Among Aphididae, Lachninae are one of the most diverse groups in host–plant association, ecological and life-history characters (Normark 2000) with males that are relatively small, but not dwarfish (in exception of the genus *Stomaphis*). Whether the parameres of Lachninae are homologous with those found in the remaining subfamilies is not clear. Because external male genitalia are composed of functionally different components (the phallus—a copulatory organ and parameres—the grasping apparatus), any unique combination of these components should be species-specificity (Song 2009). Lachninae differ from other aphids, as many species may infest the same host plant. Thus, the highly variable genitalia of Lachninae could play an important role in the context of sexual selection. According to the molecular studies of Ortiz-Rivas and Martinez-Torres (2010), Lachninae constitute an independent and basal lineage in Aphididae. However, both Heie and Węgierek (2009a) and Quednau (2010) stated that Aiceoninae, Greenideinae, Aphidinae, and Lachninae constitute a complex of modern aphids. Our previous study (Wieczorek et al. 2011) showed that Aphidinae are characterized by parameres that are not modified and are similar to those in the drepanosiphine aphids, whereas parameres of Lachninae (except for the genera *Lachnus* and *Pterochloroides*) resemble those in Aiceoninae and Greenideinae.

#### External genitalia—hypothetical plesiomorphic condition and its modifications

The contribution of comparative morphology of male external genitalia, if studied alone, is limited and needs further morphological characters, when reconstructing the phylogeny. In addition, in the absence of data in the fossil material, it is difficult to speculate which external genitalia characters should be interpreted as primitive (plesiomorphic) and which are derived. Aphids are characterized by the ‘mosaic’ type of evolution—some features have evolved independently, several times, in various evolutionary lines, which makes the interpretation of data complicated (Heie and Węgierek 2009a; Quednau 2010). Probably, the hypothetical plesiomorphic condition of the external male genitalia of aphids is characterized by the rather large, lobate and setose parameres positioned at the anterior end of the genital area as well as well developed the basal part of the phallus which protects the aedeagus and is supported by the sclerotization. This type of external genitalia is found in most modern taxa of drepanosiphine aphids and Aphidinae. In dwarfish males of Anoeciinae, Thelaxinae, Hormaphidinae and Eriosomatinae the miniaturization of the body size affects on the modification of genitalia—mostly parameres. Various modified parameres found in males of these four subfamilies do not show any strong similarites with each other (single structure in Anoeciinae and Thelaxinae, very small and barely visible in Hormaphidinae or reduced to elongated projections supported by additional sclerotization in Eriosomatinae) and have developed in different way. It suggests that reduction in the size of sexual generation has occurred independently in a number of distinct lineages within Aphididae. Whether the parameres of mentioned dwarf males are homologus with those found in the drepanosiphine aphids and Aphidinae is not clear. The question, whether they function as the grasping apparatus, is also open. It may therefore be that the parameres are non-functional. However, the mating behavior of these aphids has never been observed. Compared with the drepanosiphine aphids and Aphidinae, the external genitalia of Lachninae (except for the genera *Lachnus* and *Pterochloroides*), Aiceoninae, and Greenideinae are more specialized, that is, parameres are divided into lobate parts ended by various-shaped projections. It is possible that the extended region of parameres perform the same function, presumably that of gripping the female, as the parameres do in other aphids. Although the character of parameres appears similar in Lachninae, Aiceoninae, and Greenideinae, these subfamilies differ greatly otherwise. It seems likely that the modification of parameres have evolved independently in Lachninae as well as Aiceoninae and Greenideinae on the other hand.

The most important characters of the external male genitalia of Aphididae, which could be discussed in conjunction with the phylogenetic relationships among the studied taxa, are compared in the Table 2.

**Table 2:** The most important characters of the external male genitalia of Aphididae

Subfamily/tribe/species	1	2	3	4	5	6	7	8	9	10	11	12	13	14	15	16	17	18	19	20	21	22	23	24	25	26	27	28	29	30	31
Mindarinae																															
*Mindarus abietinus*	+	−	−	+	+	−	−	−	−	+	+	−	−	+	−	−	−	+	−	−	+	+	−	−	+	−	+	−	+	+	−
Drepanosiphinae																															
*Drepanosiphum platanoidis*	+	−	+	−	+	−	−	−	−	+	+	−	+	−	−	−	−	+	−	−	+	+	−	−	+	−	+	−	+	−	_+_
Chaitophorinae Chaitophorini																															
*Chaitophorus populeti*	+	−	+	−	+	−	−	−	−	+	+	−	+	−	−	−	−	+	−	−	+	+	−	+	−	−	+	+	−	−	+
*Periphyllus coracinus*	+	−	+	−	+	−	−	−	−	+	+	−	+	−	−	−	−	+	−	+	−	+	−	−	+	−	+	+	−	−	+
Chaitophorinae Siphini																															
*Chaetosiphella stipae*	+	−	−	+	+	−	−	−	−	+	+	−	+	−	−	−	−	+	−	+	−	+	−	+	−	−	+	−	+	+	−
*Sipha (Rungsia) maydis*	+	−	−	+	+	−	−	−	−	+	+	−	+	−	−	−	−	+	−	+	−	−	+	+	−	−	+	−	+	+	−
*Laingia psammae*	+	−	−	+	+	−	−	−	−	+	+	−	+	−	−	−	−	+	−	+	−	+	−	+	−	−	+	−	+	+	−
Calaphidinae Calaphidini																															
*Clethrobius comes*	+	−	+	−	+	−	−	−	−	+	+	−	+	−	−	−	−	+	−	−	+	+	−	−	+	−	+	−	+	−	+
Calaphidinae Panaphidini																															
*Appendiseta robiniae*	+	−	+	−	+	−	−	−	−	+	+	−	+	−	−	−	−	+	−	−	+	+	−	+	−	−	+	−	+	−	+
*Mexicallis spinifer*	+	−	+	−	+	−	−	−	−	+	+	−	+	−	−	−	−	+	−	−	+	+	−	−	+	−	+	−	+	+	−
*Myzocallis (Lineomyzocallis) walshii*	+	−	+	−	+	−	−	−	−	+	+	−	−	+	−	−	−	+	−	−	+	+	−	−	+	−	+	−	+	−	+
*Panaphis juglandis*	+	−	+	−	+	−	−	−	−	+	+	−	+	−	−	−	−	+	−	+	−	+	−	+	−	−	+	−	+	−	+
Phyllaphidinae																															
*Phyllaphis fagi*	+	−	+	−	+	−	−	−	−	+	?	?	+	−	−	−	−	+	−	+	−	+	−	+	−	−	+	−	+	−	+
*Diphyllaphis mordvilkoi*	+	−	+	−	+	−	−	−	−	+	−	+	+	−	−	−	−	+	−	+	−	+	−	−	+	−	+	−	+	?	?
Saltusaphidinae Thripsaphidini																															
*Subsaltusaphis flava*	+	−	+	−	+	−	−	−	−	+	+	−	−	+	−	−	−	+	−	+	−	?	?	+	−	−	+	−	+	?	?
Lizeriinae																															
*Lizerius ocoteae*	+	−	+	−	+	−	−	−	−	+	+	−	+	−	−	−	−	+	−	−	+	−	+	−	+	−	+	−	+	?	?
Spicaphidinae																															
*Neuquenaphis* *edwardsi*	+	−	+	−	+	−	−	−	−	+	−	+	+	−	−	−	−	+	−	−	+	+	−	−	+	−	+	−	+	?	?
Tamalinae																															
*Tamalia* sp.	+	−	+	−	+	−	−	−	−	+	−	+	+	−	−	−	−	+	−	−	+	−	+	−	+	−	+	−	+	−	+
Parachaitophorinae																															
*Parachaitophorus yamashitai*	+	−	+	−	+	−	−	−	−	+	+	−	−	+	−	−	−	+	−	−	+	+	−	−	+	−	+	+	−	?	?
Phloeomyzinae																															
*Phloeomyzus passerini*	+	−	+	−	+	−	−	−	−	+	+	−	+	−	−	−	−	+	−	−	+	+	−	+	−	−	+	−	+	?	?
Aphidinae Aphidini																															
*Aphis pomi*	+	−	+	−	+	−	−	−	−	+	−	+	−	−	+	−	−	+	−	+	−	+	−	−	+	−	+	−	+	−	+
Aphidinae Macrosiphini																															
*Brachycaudus divaricatae*	+	−	+	−	+	−	−	−	−	+	−	+	+	−	−	−	−	+	−	−	+	−	+	−	+	−	+	−	+	+	−
*Cavariella saxifragae*	+	−	+	−	+	−	−	−	−	+	−	+	+	−	−	−	−	+	−	−	+	+	−	−	+	−	+	−	+	−	+
*Hyperomyzus pallidus*	+	−	+	−	+	−	−	−	−	+	−	+	−	+	−	−	−	+	−	−	+	−	+	−	+	−	+	−	+	?	?
*Myzus cerasi*	+	−	+	−	+	−	−	−	−	+	−	+	+	−	−	−	−	+	−	−	+	+	−	−	+	−	+	−	+	−	+
*Pterocomma populeum*	+	−	+	−	+	−	−	−	−	+	−	+	+	−	−	−	−	+	−	−	+	+	−	−	+	−	+	−	+	−	+
Hormaphidinae Nipponaphidini																															
*Neothoracaphis yanonis*	+	−	−	+	+	−	−	−	−	+	?	?	−	−	+	−	−	+	−	+	−	−	+	+	−	−	+	−	+	?	?
*Nipponaphis distyliicola*	+	−	−	+	+	−	−	−	−	+	?	?	−	−	?	−	−	+	−	+	−	−	+	?	?	−	+	−	+	?	?
*Monzenia globuli*	+	−	−	+	+	−	−	−	−	+	?	?	−	−	?	−	−	+	−	+	−	−	+	?	?	−	+	−	+	?	?
Anoeciinae																															
*Anoecia vagans*	+	−	−	+	−	+	−	−	−	+	−	−	+	−	−	−	−	+	−	−	+	−	+	+	−	−	+	−	+	+	−
Thelaxinae																															
*Glyphina betulae*	+	−	−	+	−	+	−	−	−	+	−	−	−	−	−	−	+	+	+	−	−	−	+	?	?	−	+	−	+	+	−
Eriosomatinae Eriosomatini																															
*Tetraneura (Tetraneurella) nigriabdominalis*	+	−	+	−	−	−	+	−	+	−	−	+	−	−	−	−	−	+	−	+	−	−	+	+	−	−	+	−	+	+	−
Eriosomatinae Pemphigini																															
*Pemphigus versicarius*	+	−	+	−	−	−	+	−	+	−	−	+	−	−	−	−	−	+	−	+	−	−	+	+	−	−	+	−	+	?	?
*Pachypappa tremulae*	+	−	+	−	−	−	+	−	+	−	−	+	−	−	−	−	−	+	−	+	−	+	−	+	−	−	+	−	+	?	?
*Prociphilus fraxini*	+	−	+	−	−	−	+	−	+	−	−	+	−	−	−	−	−	+	−	+	−	−	+	+	−	−	+	−	+	?	?
Greenideinae Greenideini																															
*Greenidea (Trichosiphum) carpini*	+	−	+	−	−	−	−	+	−	+	+	−	−	−	+	−	−	+	−	−	+	−	+	+	−	−	+	−	+	?	?
*Eutrichosiphum sinense*	+	−	+	−	−	−	−	+	−	+	+	−	−	−	−	+	−	+	−	+	−	−	+	+	−	−	+	−	+	?	?
*Mollitrochosiphum (Metatrichosiphon) yamabiwae*	+	−	+	−	−	−	−	+	−	+	+	−	+	−	−	−	−	+	−	−	+	−	+	+	−	−	+	−	+	?	?
Cervaphidini																															
*Anomalosiphum mendeli*	+	−	+	−	−	−	−	+	−	+	+	−	−	−	+	−	−	+	−	+	−	−	+	+	−	−	+	−	+	?	?
Schoutedeniini																															
*Schoutedenia ralumensis*	+	−	+	−	−	−	−	+	−	+	+	−	−	−	−	+	−	+	−	+	−	−	+	?	?	−	+	−	+	?	?
Aiceoninae																															
*Aiceona japonica*	+	−	+	−	−	−	−	+	−	+	−	+	−	−	−	+	−	+	−	−	+	−	+	−	+	−	+	−	+	?	?
Lachninae Lachnini																															
*Lachnus pallipes*	−	+	+	−	+	−	−	−	−	+	−	+	−	−	+	−	−	+	−	+	−	+	−	+	−	−	+	−	+	+	−
*Pterochloroides persicae*	+	−	+	−	+	−	−	−	−	+	−	+	+	−	−	−	−	+	−	+	−	+	−	+	−	−	+	−	+	?	?
*Stomaphis aceris*	+	−	+	−	−	−	−	+	−	+	−	+	−	−	−	+	−	+	−	+	−	+	−	+	−	−	+	−	+	?	?
*S. quercus*	+	−	+	−	−	−	−	+	−	+	−	+	+	−	−	−	−	+	−	+	−	+	−	+	−	−	+	−	+	+	−
*Maculolachnus submacula*	+	−	+	−	−	+	−	−	−	+	−	+	−	−	−	+	−	+	−	−	+	+	−	−	+	−	+	−	+	+	−
*Schizolachnus piniradiatae*	+	−	+	−	−	−	−	+	−	+	−	+	+	−	−	−	−	+	−	+	−	+	−	−	+	+	−	+	−	?	?
Lachninae Eulachnini																															
*Eulachnus agilis*	+	−	+	−	−	−	−	+	−	+	+	−	+	−	−	−	−	+	−	+	−	+	−	−	+	+	−	+	−	?	?
*Cinara cedri*	+	−	+	−	−	−	−	+	−	+	+	−	+	−	−	−	−	+	−	+	−	+	−	−	+	+	−	+	−	?	?
*C. piceicola*	+	−	+	−	−	−	−	+	−	+	−	+	+	−	−	−	−	+	−	−	+	+	−	−	+	+	−	+	−	?	?
*C. (Cedrobium) laportei*	+	−	+	−	−	−	−	+	−	+	+	−	+	−	−	−	−	+	−	+	−	+	−	−	+	+	−	+	−	?	?

*1* Parameres positioned above the basal part of the phallus, *2* parameres positioned laterally to the basal part of the phallus, *3* parameres large, *4* parameres small, *5* parameres not modified, *6* parameres modified into single structure, *7* parameres modified into elongated projections, *8* parameres modified into lobate parts ended by projections, *9* additional sclerotization of parameres projections present, *10* additional sclerotization of parameres projections absent, *11* parameres fused basally, *12* parameres separate, *13* parameres covered by setae on the whole surface, *14* parameres covered by setae on the inner margin, *15* parameres covered by setae on the outer margin, *16* parameres covered by setae on the lobate parts, *17* parameres covered by setae on the projections, *18* basal part of the phallus present, *19* basal part of the phallus strongly reduced, *20* basal part of the phallus short, *21* basal part of the phallus long, *22* basal part of the phallus with setae, *23* basal part of the phallus without setae, *24* sclerotized arms of a similar length to the proximal and distal parts, *25* sclerotized arms: proximal part short and wide, distal part long and thin, *26* distal parts of sclerotized arms with the upper half-circle-shaped structure, *27* distal parts of sclerotized arms without the upper half-circle-shaped structure, *28* distal parts of sclerotized arms with rounded processes, *29* distal parts of sclerotized arms without rounded processes, *30* aedeagus short, *31* aedeagus long

*+* Character present, *−* character absent, *?* character unknown

## Summary

The males of most aphids, unlike the males of other Hemiptera, do not appear until autumn (or even early in the summer) and have to mature quickly. Their mating behavior characterized by many short copulations requires a certain degree of simplicity in the structure of the reproductive system—both external and internal. The external male genitalia of Aphididae are well developed and typically consist of the phallus, which is composed of the sclerotized basal part with its articulation and a membranous apical part—the aedeagus as well as parameres. This state probably represents the hypothetical plesiomorphic condition of the external male genitalia of aphids. The phallus structure presents a stable character among all of the subfamilies studied, whereas parameres appeared to be highly variable between the different subfamilies studied (the most varied in Lachninae). They are observed in taxa with normal-sized (Aiceoninae, Greenideinae and Lachninae) and dwarfish males (Anoeciinae, Eriosomatinae and Thelaxinae) and could be useful in considerations of the relationships and phylogeny of aphids. However, the homology of non-modified and modified structures of parameres is not clear. In addition, both the phallus and parameres show the greatest variability in their form and number of setae and may provide characters of taxonomic and diagnostic importance.
